# A Comprehensive Analysis on Spread and Distribution Characteristic of Antibiotic Resistance Genes in Livestock Farms of Southeastern China

**DOI:** 10.1371/journal.pone.0156889

**Published:** 2016-07-07

**Authors:** Na Wang, Xinyan Guo, Zheng Yan, Wei Wang, Biao Chen, Feng Ge, Boping Ye

**Affiliations:** 1 Nanjing Institute of Environmental Sciences, Ministry of Environmental Protection of China, Nanjing, 210042, PR China; 2 Key Laboratory of Pesticide Environmental Assessment and Pollution Control, Ministry of Environmental Protection of China, Nanjing, 210042, PR China; 3 School of Life Science and Technology, China Pharmaceutical University, Nanjing, 210009, PR China; 4 Chinese Society For Environmental Sciences, Beijing, 100082, PR China; Nankai University, CHINA

## Abstract

The pollution of antibiotic resistance genes (ARGs) in livestock farms is a problem which need to be paid more attention to, due to the severe resistance dissemination and the further human health risk. In this study, all the relevant exposure matrices (manure, soil and water) of sixteen animal farms in Southeastern China were sampled to determine twenty-two ARGs conferring resistance to five major classes of antibiotics including tetracyclines, sulfonamides, quinolones, aminoglycosides, and macrolides. The results showed that the spread property of *sul* genes was most extensive and strong, followed by *tet* and *erm* genes. The abundance of *tet* genes expressing ribosomal protection proteins (*tetM*, *tetO*, *tetQ*, *tetT* and *tetW*) was higher than that expressing efflux pump proteins (*tetA*, *tetC*, *tetE* and *tetG*) in each type of samples. The high abundance and frequency of *ermB* gene in the matrices should be paid more attention, because macrolides is a major medicine for human use. For manures, it was found that the similar ARGs distribution rules were existing in poultry manure or porcine manure samples, despite of the different origins of these two types of livestock farms. Meanwhile, it was interesting that the distribution rule of *tet* genes in animal manure was nearly the same as all the ARGs. For soils, the result of nonmetric multi-dimensional scaling (NMDS) analysis showed that the pollution of ARGs in the soils fertilized by poultry and cattle manures were more substantial in northern Jiangsu, but no significant ARGs diversity was observed among porcine manured soils of five different regions. Furthermore, most ARGs showed significant positive relationships with environmental variables such as concentration of sulfonamides, tetracyclines, Cu, Zn and total organic carbon (TOC). The pollution profile and characteristics of so many ARGs in livestock farms can provide significative foundation for the regulation and legislation of antibiotics in China.

## Introduction

Veterinary antibiotics are widely used in many countries to treat diseases and promote animal growth. The large amounts of antibiotics applicated for production animals have selected for plenty of antibiotic-resistant bacteria (ARB) predominantly in the gastrointestinal tracts of animals, making animal manure a potential hotspot of antibiotic resistance genes (ARGs) [1]. After the land application of antibiotic-polluted manure in agricultural practice, the horizontal transfer of ARGs from fecal microorganisms to indigenous environmenatal bacteria is an important factor in resistance dissemination [2]. Furthermore, antibiotics released by animal manure can provide a positive selective pressure for ARBs. The World Health Organization (WHO) has identified ARGs as one of the most critical human health challenges in the next century [3], due to the fact that some ARBs in soil and manure are phylogenetically close to human pathogens, making genetic exchange more likely [4].

Recently, antibiotic, ARBs and ARGs have been reported to distribute extensively across various environmental matrices. From the catchment scale, diverse ARGs were found in the water or sediment phase of rivers in several countries [5–10], among which sulfonamide resistance genes and tetracycline resistance genes were the most prevalent genes, however, the prevalence of macrolide resistance genes became higher than most of *tet* genes [5]. The concluding remarks of the studies above were that intensive human activities are one of the most important factors affecting the dissemination of ARGs. Livestock farming is one of the main human activities, which contribute more for the ARGs selection and dissemination [11]. From the livestock farms scale, most reports have concentrated on the fate and remove efficiency of antibiotics and ARGs in the wastewater treatment systems [12–15], indicating that some ARGs were significantly increased after an aerobic process. Only a few studies have focused on the abundance and diversity of ARGs in soil adjacent to livestock feedlots [1, 3], in which the studying objects were mainly tetracycline and sulfonamide resistance genes. However, macrolides, fluoroquinolones, aminoside and cephalosporin were often applied in livestock farms of China frequently and largely, the occurrence of which in soils fertilized by wastewater were observed in several studies [16, 17], but the prevelence and distribution of the corresponding ARGs in each exposure matrices of the livestock farms are rare, such as manure, soil and water bodies. Recently, it is necessary to obtain the systematic analysis on the spread and distribution characteristic of most ARGs in all the exposure matrices of livestock farms, which can provide basic pollution status for the regulation and legislation of antibiotics in China.

Based on a recent survey [18], the total antibiotic usage in China for 2013 was estimated to be approximately 162000 tons, of which animal consumption accounted for about 52%, while the animal usage in UK for 2013 was 420 tons, and that in USA for 2011/2012 was 14600 tons. It should be paid more attention to Southeastern China because the animal husbandry is developed here due to the proximity to the Yangtze River Basin and the rich resources of water. For Jiangsu Province, the overall output of livestock was 4.19 ×10^6^ tons in 2013, and the output of pork was 2.30×10^6^ tons and 2.32×10^6^ tons in 2013 and 2014, respectively. Accroding to the investigation by Zhang et al. [18], Yangtze River Downstream basins received relatively higher discharge of the target antibiotics and showed the higher emission densities. Therefore, it is important to recognize the pollution profile and characteristic of antibiotics and its ARGs in the livestock farms of Southeastern China, which can help to obtain the overall pollution status of China.

To obtain the overall pollution profile and spread characteristics of ARGs in Southeastern China, all the relevant exposure matrices of sixteen animal farms in Jiangsu Province were sampled to determine twenty-two ARGs conferring resistance to five major classes of antibiotics including tetracyclines, sulfonamides, quinolones, aminoglycosides, and macrolides in this study. Based on the data, dominant ARGs in manure, soil and water were screened, and the spead characteristics of different ARGs can be further analyzed. By the non-parametric test and nonmetric multidimensional scaling (NMDS) plots, the animal-type related variance of ARGs distribution in manures and the spatial variance of ARGs distribution in manured soils were statistically calculated. Furthermore, the influencing factors on the abundance of ARGs, including antibiotics, heavy metals, and some physicochemical parameters, were analyzed by pearson correlation and redundancy analysis (RDA) analysis. Anyway, this study systematically showed the pollution profile and characteristics of so many ARGs to five classes of frequently uesd veterinary antibiotics in Jiangsu Province of China, which can provide significative foundation for the regulation and legislation of antibiotics in China.

## Materials and Methods

### Sample collection

A total of 53 manure samples, 50 amended soil samples and 28 water samples (11 surface water samples, 17 wastewater samples) were collected for investigation from 16 animal farms located in Nanjing (NJ), Changzhou (CZ), Suqian (SQ), Xvzhou (XZ), and Haian (HA) cities in Jiangsu Province between March-June 2014, including 5 swine, 6 chicken, and 5 cattle livestock farms (S1 Table). The study was permitted and approved by the Ministry of Environmental Protection, China. The land accessed was not privately owned or protected. No protected species were sampled. Manure samples were taken from daily fresh excrement heaps. For those farms that produce organic fertilizers through composting, fertilizer samples were also taken. Soil samples were obtained from the top 0 to 15 cm of the manure applied arable lands adjacent to corresponding livestock farms. Flushing wastewater in farms and surface water in ponds and lakes adjacent to farms were sampled too. For each sampling site, three subsamples were collected using a multiple-point sampling method and then were mixed together to obtain the composite samples. Each sample was placed into a plastic container and immediately chilled in an icebox. All samples were transported under cool conditions to the laboratory, and then stored in the dark at −20°C until DNA extraction (within 1 week).

### DNA extraction

For manure samples, DNA was extracted using Stool DNA Kit (Omega D4015-02, Norcross, GA, USA) according to the manufacture’s protocol. For soil samples, DNA was obtained employing PowerSoil DNA Isolation Kit (MoBio Laboratories, Carlsbad, CA, USA) following the manufacturer's protocol. For water samples, they were firstly filtrated with sterilized filter paper to separate undissolved substance, then filtrated with 0.22 μm nitrocellulose filter membrane (Millipore, Boston, USA) to intercept bacterium. Total volume of water samples processed was recorded for the following analysis. All filter papers and membranes were stored at −80°C. Total DNA was extracted from the sterilized filter papers together with the nitrocellulose filter membranes prepared previously using a Water DNA Kit (OMEGA, Norcross, GA, USA) according to manufacturer’s instructions. The concentration and quality of the extracted DNA were determined by spectrophotometer analysis and 1% agarose gel electrophoresis.

### Primer design

The ARGs, including nine tetracycline resistance genes (*tetA*, *tetC*, *tetE*, *tetG*, *tetM*, *tetO*, *tetQ*, *tetT* and *tetW*), three sulfonamide resistance genes (*sul1*, *sul2* and *sul3*), three quinolone resistance genes (*oqxB*, *qnrS*, and *qnrD*), two macrolide resistance genes (*ermB* and *ermC*), three aminoglycoside resistance genes (*aph*, *aadD*, and *aac*), and two multidrug resistance genes (*acrA* and *acrB*) were analyzed in this study. Complete accession numbers of DNA sequences were obtained from NCBI gene database, according to which the primers were designed by Primer 5.0 (S2 Table). The specificity of the primers was tested by Primer-BLAST (http://blast.ncbi.nlm.nih.gov/Blast.cgi), after which the primers were synthetized by the biological company.

### Construction of qPCR standards

Amplification of the 22 ARGs and 16S rRNA were performed in PCR machine (Bio-Rad, CA, USA). The PCR mixture (25 μL total volume) contained 1× PCR buffer, 2.0 mmol/L MgCl2, 1.0 mol/L dNTP mixture, 400 nmol/L each primer, 1.25 U of Taq DNA polymerase, and 100 ng template DNA. The temperature program was initially denaturated in a thermal cycler for 5 min at 94°C, then subjected to 35 amplification cycles of 30 s at 94°C, 30 s at different annealing temperatures and 45 s at 72°C, followed by a final extension step of 6 min at 72°C. The PCR products were analyzed by electrophoresis on a 1.5% agarose gel. After the PCR products carrying each ARGs were separated by 2% agarose gel electrophoresis, they were purified by an EasyPure Quick Gel Extraction Kit (TransGen Biotech, China). The purified PCR products were ligated into pEasy-T3 vector (TransGene Biotech, China) and then cloned into Trans1-T1 phage resistant chemically competent cell (TransGen Biotech, China). Positive clones were screened by PCR to verify cloning of the target genes and sequenced. The sequences were analyzed by the BLAST alignment tool (http://www.ncbi.nlm.nih.gov/blast/). Positive clones with the target gene were chosen as the standards for real-time qPCR. Plasmids carrying the target genes were extracted using a Plasmid Miniprep Kit (Axygen, USA) and further used to prepare the standardized product for real-time PCR.

### Quantitative PCR

Real-time qPCR was applied to quantify ARGs and 16S rRNA gene in DNA extracted from samples. The qPCR reactions were conducted in 96-well plates. All real-time qPCR assays were performed in triplicate using the 2× UItraSYBR Mixture (CWBIO, China) on the CFX96 Touch™ Real-Time PCR Detection System (Bio-Rad, USA). The real-time qPCR program was as follow: initial denaturing at 95°C for 10min, followed by 40 cycles of 10 s at 95°C, 30 s at different annealing temperatures, and 30 s at 72°C. The fluorescence data were acquired at 72°C, and the final melting curve was constructed with temperature ramping up from 65 to 95°C. Tenfold serial diluted calibration curve of each ARG was tested in triplicate on the same PCR plate. For all standard curves, the linear coefficients (R^2^) were greater than 0.990, and their amplification efficiencies were between 95% and 105%. The equations of standard curves were listed in S3 Table.

### Antibiotic, metal, Total organic carbon (TOC) and pH determination

Concentrations of tetracyclines (tetracycline, oxytetracycline, chlortetracycline and doxycycline), sulfonamides (sulfadiazine, sulfamerazine, sulfamethazine, sulfadimethoxine, sulfamethoxazole, sulfathiazole, sulfachloropyridazine), quinolones (norfloxacin, ciprofloxacin, enrofloxacin, ofloxacin, fleroxacin, sarafloxacin) and macrolides (erythromycin, roxithromycin, tylosin and josamycin) were analyzed with the published method by Guo et.al. [19]. Eight metal elements, i.e. chromium (Cr), manganese (Mn), cobalt (Co), nickel(Ni), copper (Cu), zinc (Zn), cadmium (Cd) and lead (Pb), were determined by inductively coupled plasma-mass spectrometry (ICP-MS, Agilent 7500, Agilent, USA) [20]. Quantification for TOC and pH of each sample were carried out by using organic carbon analyzer (Analytikjena, Germany) and pH meter (HQ30d, HACH, USA), respectively.

### Statistics

Averages and standard errors were determined using SPSS for Windows version 19.0. Nonparametric Kruskal-Wallis tests and Shapiro-Wilk tests were conducted to evaluate the statistical significance of difference with P value < 0.05. Clustering analysis was performed using HemI software (version 1.0.1, Heatmap Illustrator). Bray-Curtis coefficients were calculated by PAST 3.0 software for each of the samples using the natural log-transformed data of absolute concentration of each ARG. Redundancy Analysis (RDA) was performed with Canoco for Windows (Version 4.5).

## Results and Discussion

### Dominant ARGs in manure, soil and water

Twenty-two ARGs conferring resistance to five major classes of antibiotics including tetracyclines, sulfonamides, quinolones, aminoglycosides, and macrolides were detected in animal manures, manured soils samples, wastewater and surface water of the sampling feedlots. The prevalence of ARGs in wastewater and surface water was prescribed in the previous paper by Chen et. al [15]. It was found that the ARGs were found frequently and extensively in the livestock farm environment. In manure, *ermB*, *tetM* and *sul2* were detected in the highest concentration and frequency, with the mean level above 10^9^copies/g and detection frequency higher than 90% (see S1 Fig). In soil, *tet* and *sul* genes were the most dominant genes, the absolute concentration of which were higher as 2–4 orders of magnitude than that of the other investigated ARGs (see S2 Fig). *sul2* was the gene with the highest concentration of 1.73×10^9^copies/g, followed by *tetC*, *tetM* and *sul1*, with the concentration higher than 10^8^copies/g.

**Fig 1:**
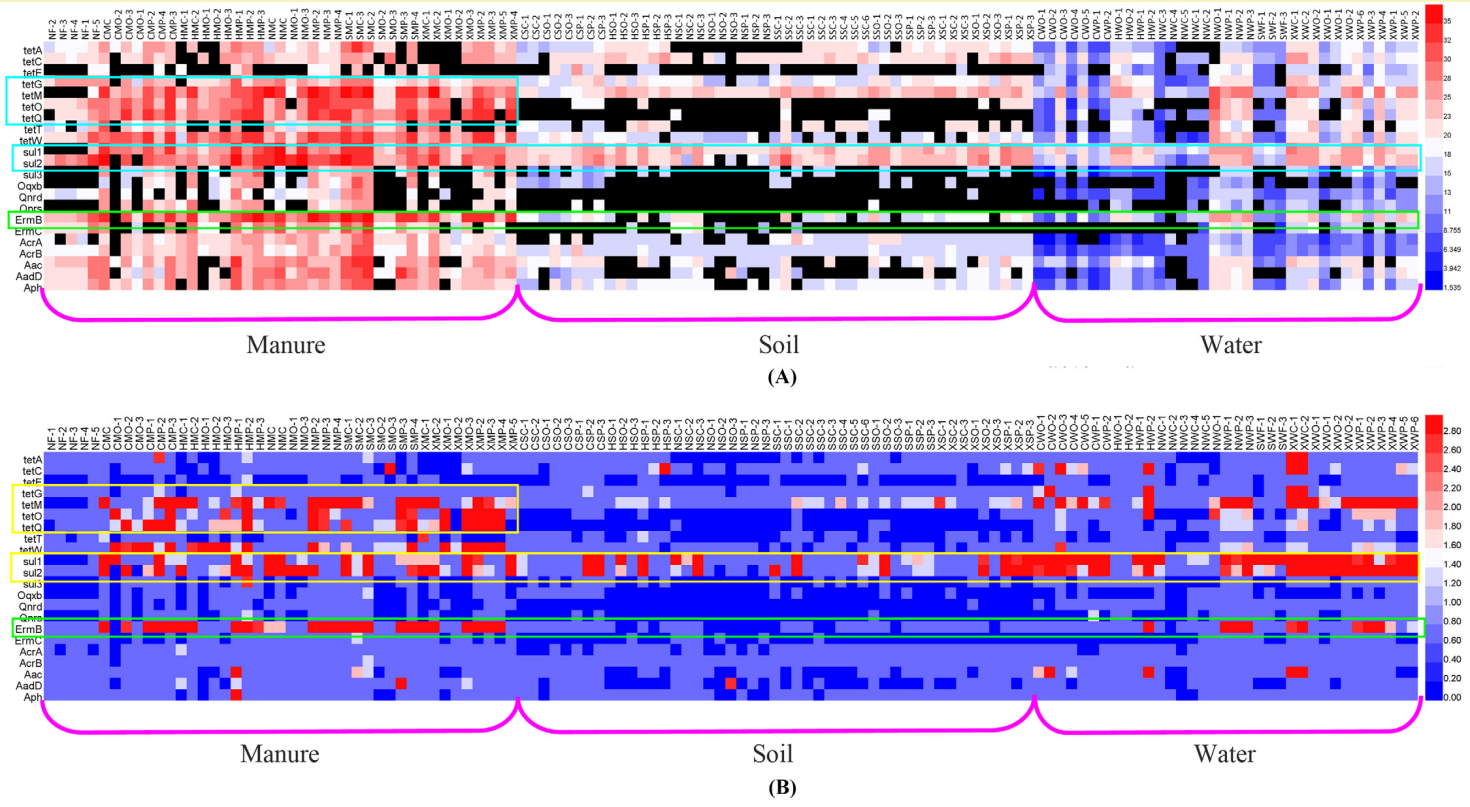
**Heatmap of the absolute (A) and relative (B) concentration of the targeted ARGs in manure, soil and water samples.** (Columns show the ARGs concentrations, and red indicates an increase in gene abundance, whereas blue indicates a decrease. No change corresponds to 0 in the color scale.)

Overall, from the heatmap of the absolute abundance (gene copy numbers of ARGs in 1g soil or 1 mL water) and relative abundance (gene copy numbers of ARGs normalized to the gene copy numbers of 16S rRNA) of the targeted ARGs in manure, soil and water samples (see Fig 1), we can firstly see that *sul1* and *sul2* genes were the most abundant and frequent genes detected in all the matrices, reflecting their extensive spread nature. It stayed well in step with the research of Fahrenfeld et.al. that the dissipation rates of *sul1* and *sul2* were slower in manured soils, explaining why *sul1* and *sul2* were detected in background samples [21]. Furthermore, another research found that the concentration of *sul* genes in soil increased after 289 days from the fertilization of sulfonamides contained manure [22]. Therefore, it can be obtained that the persistence of *sul* genes in environment may attribute to either slow attenuation rates or rapid growth/selection of bacteria carrying *sul1* and *sul2* or an increase in horizontal gene transfer for these genes.

Secondly, *tet* genes were also abundant in manure samples, but not as high in the soil and water as in the manure, indicating that the proliferation and dissemination of *tet* genes were weaker than that of *sul* genes. For *tet* genes, the abundance of these expressing ribosomal protection proteins (*tetM*, *tetO*, *tetQ*, *tetT* and *tetW*) was higher than the ones expressing efflux pump proteins (*tetA*, *tetC*, *tetE* and *tetG*) in each type of samples, showing that ribosomal protection proteins can endow bacteria the powerful tetracyclin-resistance. According to the published reports [21], *tetO* and *tetW* were significantly higher in post- versus pre-manured soil, but no effects on *tetG* soil concentration was observed despite the presence of it in applied manure. It can be seen that the transport of bacteria hosting *tet* genes expressing efflux pump proteins or horizontal gene transfer for them were not prevelant.

Thirdly, it is noted that that *ermB* gene was most abundant and frequent in the matrices, but macrolides is a major medicine for human use, which is a type of broad-spectrum antibiotic sensitive to Gram-positive bacterium and *Mycoplasma*, playing an important role in substituting for penicillin clinically. However, macrolides is now a veterinary medicine applied in livestock generally, including erythromycin, roxithromycin, tylosin, josamycin, clindamycin. Jensen et.al. have isolated 9 strains of macrolide-resistant Gram-positive bacterium, 8 strains of which carried *ermB* gene [23]. According to the published paper, the detection frequency of *ermB* in isolated *Staphylococcus* was 17.1% in clinical, while that of *ermC* was 0.66%. The veterinary use of macrolides may contribute significantly to the high antibiotic-resistance frequency in clinical bacteria. Furthermore, the extensive detection of aminoglycoside-resistant genes (*aph*, *aadD*, and *aac*) in soils was also a problem similar with macrolides, due to the clinical use.

### Variance of ARGs distribution in manures

**Fig 2:**
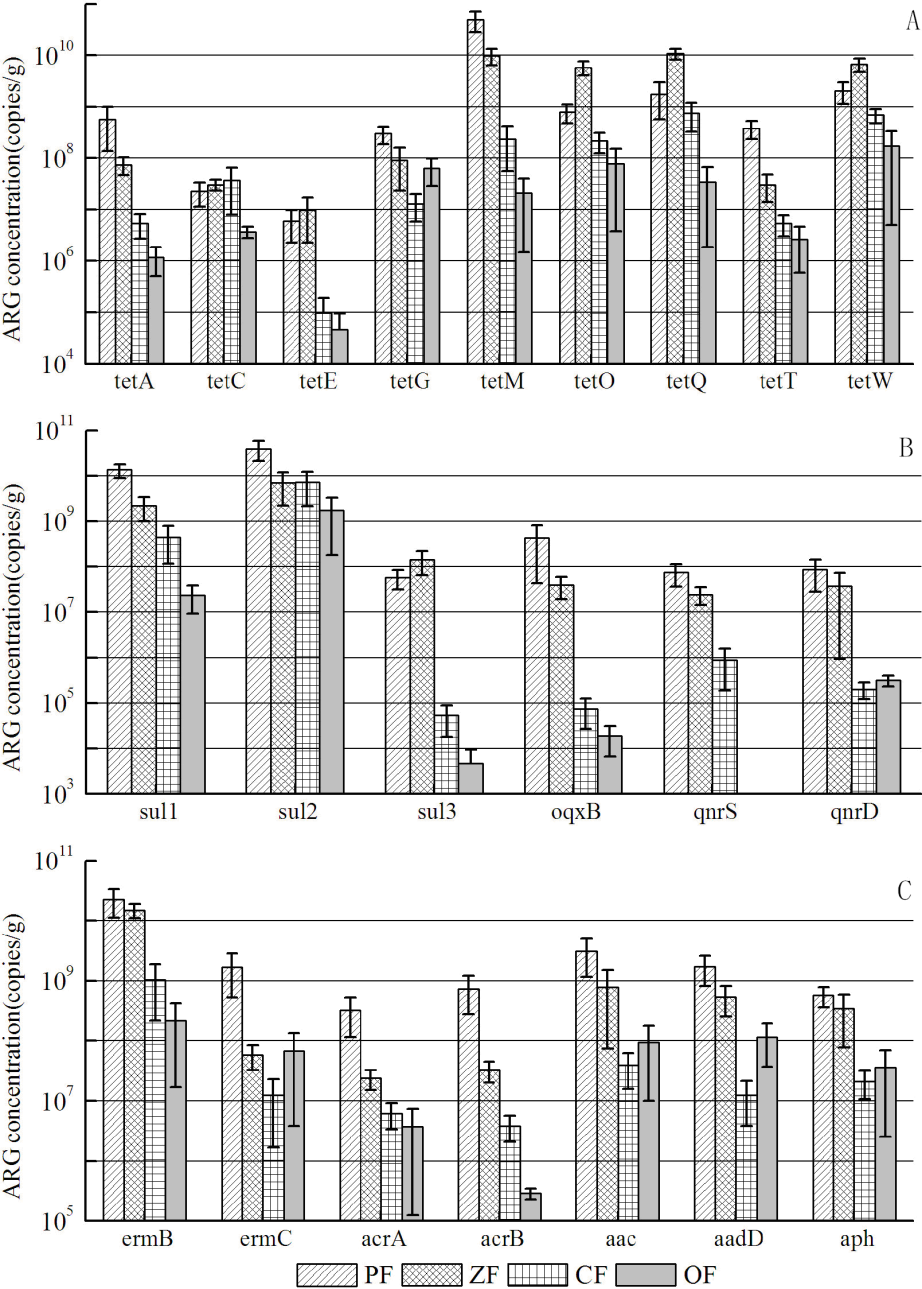
Variance of each ARGs in poultry manure (PF), porcine manure(ZF), cattle manure (CF) and organic fertilizer(OF). (*tet*: *tetA*, *tetC*, *tetE*, *tetG*, *tetM*, *tetO*, *tetQ*, *tetT*, *tetW; sul*: *sul1*, *sul2*, *sul3; qnr*: *oqxB*, *qnrS*, *qnrD; erm*: *ermB*, *ermC; acr*: *acrA*, *acrB; ami*:*aac*, *aadD*, *aph*)

**Fig 3:**
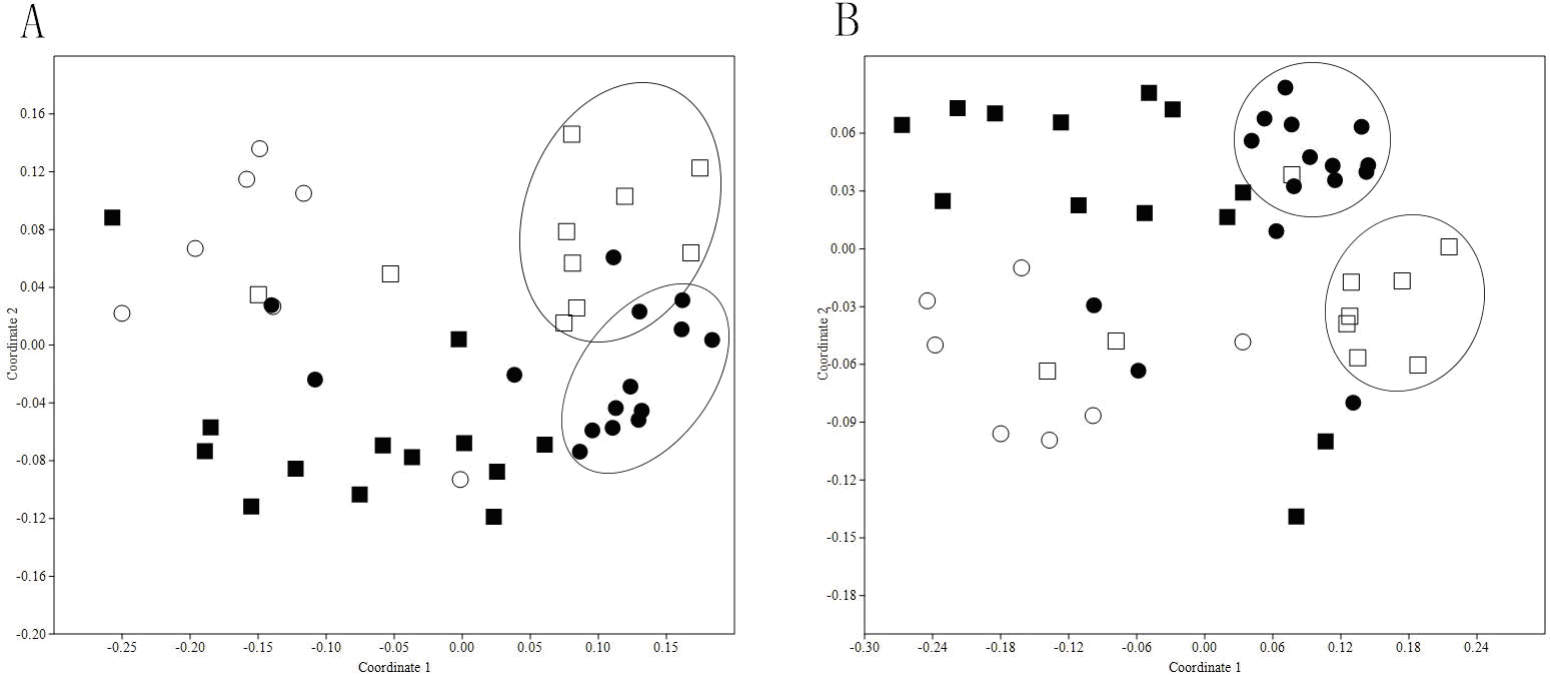
**NMDS plots of absolute abundance of *tet* genes (A) and all the ARGs (B).** (○:organic fertilizer; ●:porcine manure; □:poultry manure; ■:cattle manure) (Bray-Curtis similarity coefficients were calculated from absolute abundance of ARGs and plotted in a multidimensional scaling graph. The distance between points indicates the degree of difference in the diversity of ARGs between samples)

Through the non-parametric test, there was no significant difference of ARGs average levels in different regions, reflecting that the location of the livestock farms had no effect on ARGs distribution in animal manures. It was possibly attributed to the fact that the use patterns of antibiotics in livestock farms were nearly the same as in Jiangsu Province of China. However, the significant difference was observed in the ARG levels concerning different animal types. Except *tetG*, *tetO*, *tetW* and *sul2*, the other ARG levels in poultry and porcine manures were higher than that of cattle manure and organic fertilizer (see Fig 2), which can be explained by the higher antibiotic usage in poultry and porcine farms than that in cattle farms. A NMDS plot of absolute abundance of ARGs showed the diversity of ARGs converged in manure samples, which can also indicate that ARG concentrations in poultry and porcine manures had significant variance with that in cattle manure and organic fertilizer (see Fig 3). In the livestock farm, compost was a useful approach to treat animal manures, which can be produced as commercial organic fertilizer. It was reported that *tet* gene levels can be decreased through compost [24]. In this study, we can see the ARG levels were lower in organic fertilizer, which can strengthen the statement that compost is a helpful way to eliminate ARGs in animal manures. From Fig 3, it was clear that there were two clusters of points separately from the other points, which represented poultry manure and porcine manure samples respectively, indicating that the similar ARGs distribution existed in poultry manure or porcine manure samples, despite of the different origins of these two types of livestock farms. Meanwhile, it was interesting that the distribution pattern of *tet* genes in animal manure was nearly the same as all the ARGs. So *tet* genes can be considered as the indicative genes in animal manure.

### Variance of ARGs distribution in manured soils

**Fig 4:**
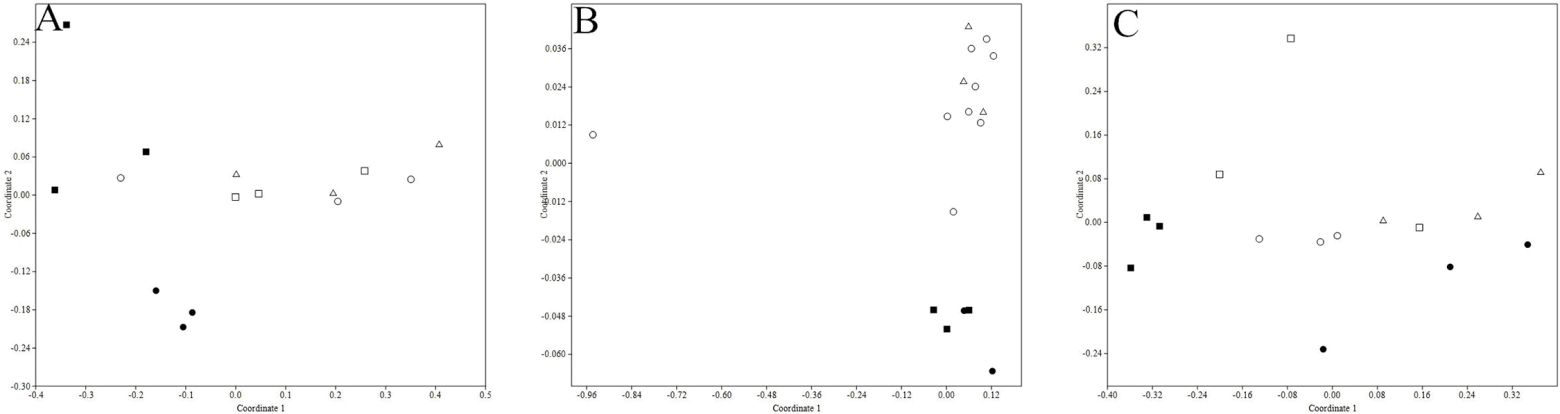
**Distribution characteristics of ARGs in soils of cattle farms (A), poultry farms (B) and porcine farms (C) located in five different regions.** (○: SQ ●: CZ □: HA ■: NJ △: XZ)(Bray-Curtis similarity coefficients were calculated from absolute abundance of ARGs and plotted in a multidimensional scaling graph. The distance between points indicates the degree of difference in the diversity of ARGs between samples)

According to the degree of economic development, Jiangsu Province can be roughly divided into three regions, northern Jiangsu (SQ, XZ, HA), southern Jiangsu (CZ) and central Jiangsu (NJ). The result of NMDS analysis showed that ARGs distribution rules in soil samples adjcent to poultry livestock farms of SQ and XZ were different from the other sampling regions. From Fig 4B, relatively speaking, the pollution levels of ARGs in SQ and XZ were more serious (stress < 0.2). Concerning the soils fertilized by cattle manures, the pollution levels of ARGs were more serious in northern Jiangsu, including SQ, XZ and HA, which was shown in Fig 4A (stress < 0.2). However, no significant ARGs diversity was observed among porcine manured soils of five different regions (Fig 4C, stress < 0.2).

**Fig 5:**
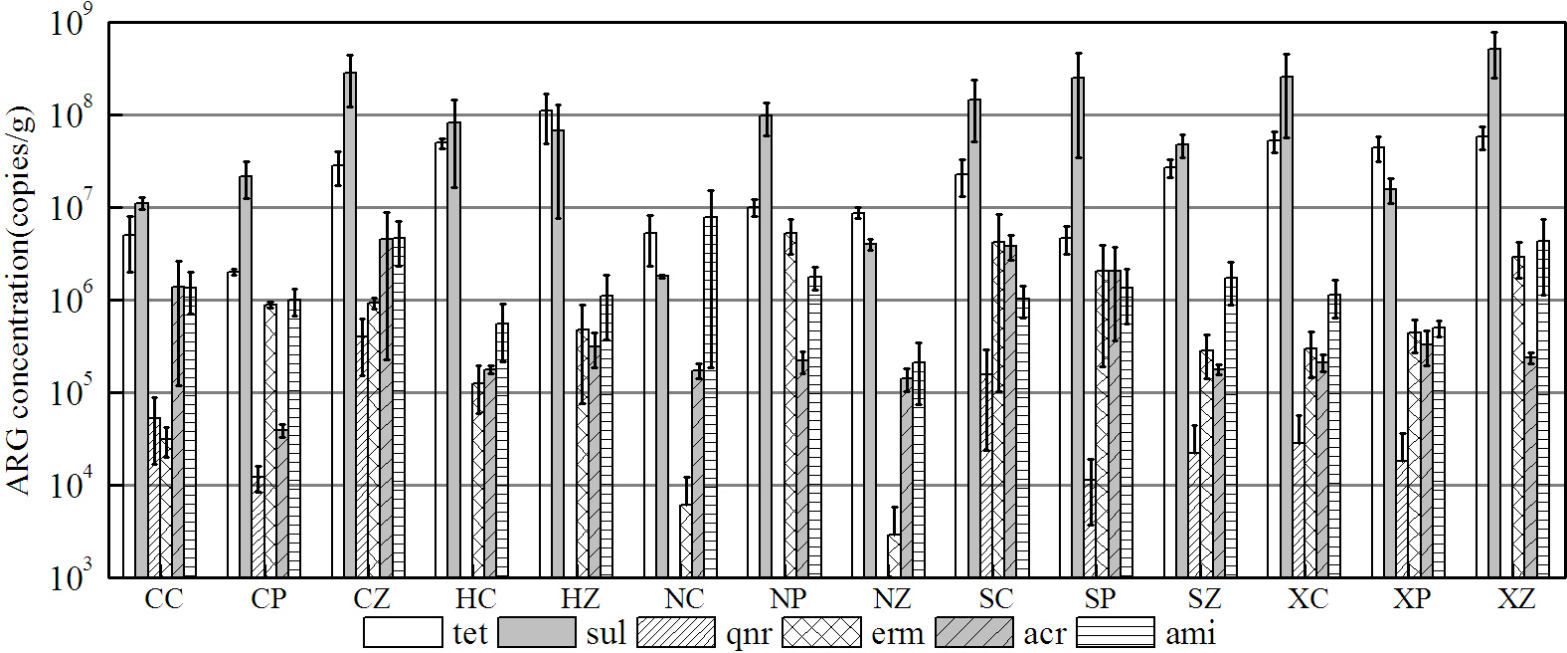
Abundance of 6 classes of ARGs in manured soils of different livestock farms. (First letter: C-CZ, H-HA, N-NJ, S-SQ, X-XZ; Second letter: Z-porcine manure; P-poultry manure; C-cattle manure. *tet*: *tetA*, *tetC*, *tetE*, *tetG*, *tetM*, *tetO*, *tetQ*, *tetT*, *tetW; sul*: *sul1*, *sul2*, *sul3; qnr*: *oqxB*, *qnrS*, *qnrD; erm*: *ermB*, *ermC; acr*: *acrA*, *acrB; ami*:*aac*, *aadD*, *aph)*

ARGs distribution in soils also depended on the animal type of the livestock farms. From Fig 5, the abundance of each ARGs was higher in porcine manured soils than that in the other animal types (p<0.05), especially *tet* and *sul* genes for CZ. For HA, there was no significant difference of ARG levels between porcine manured and cattle manured soils (p > 0.05). For Nanjing, the *sul* and *erm* genes concentrations were the highest in poultry manured soils, while *acr* genes were detected at the highest level in cattle manured soils. For SQ, the detection levels had no significant difference among all the ARGs, except for *qnr* genes, the levels of which were the highest in cattle manured soils. For XZ, the abundance of *sul* genes was lower in poultry manured soils than that in the other livestock farms (P<0.05), and no *qnr* genes were found in the porcine manured soils here.

#### Influence factors on the abundance of ARGs

**Fig 6:**
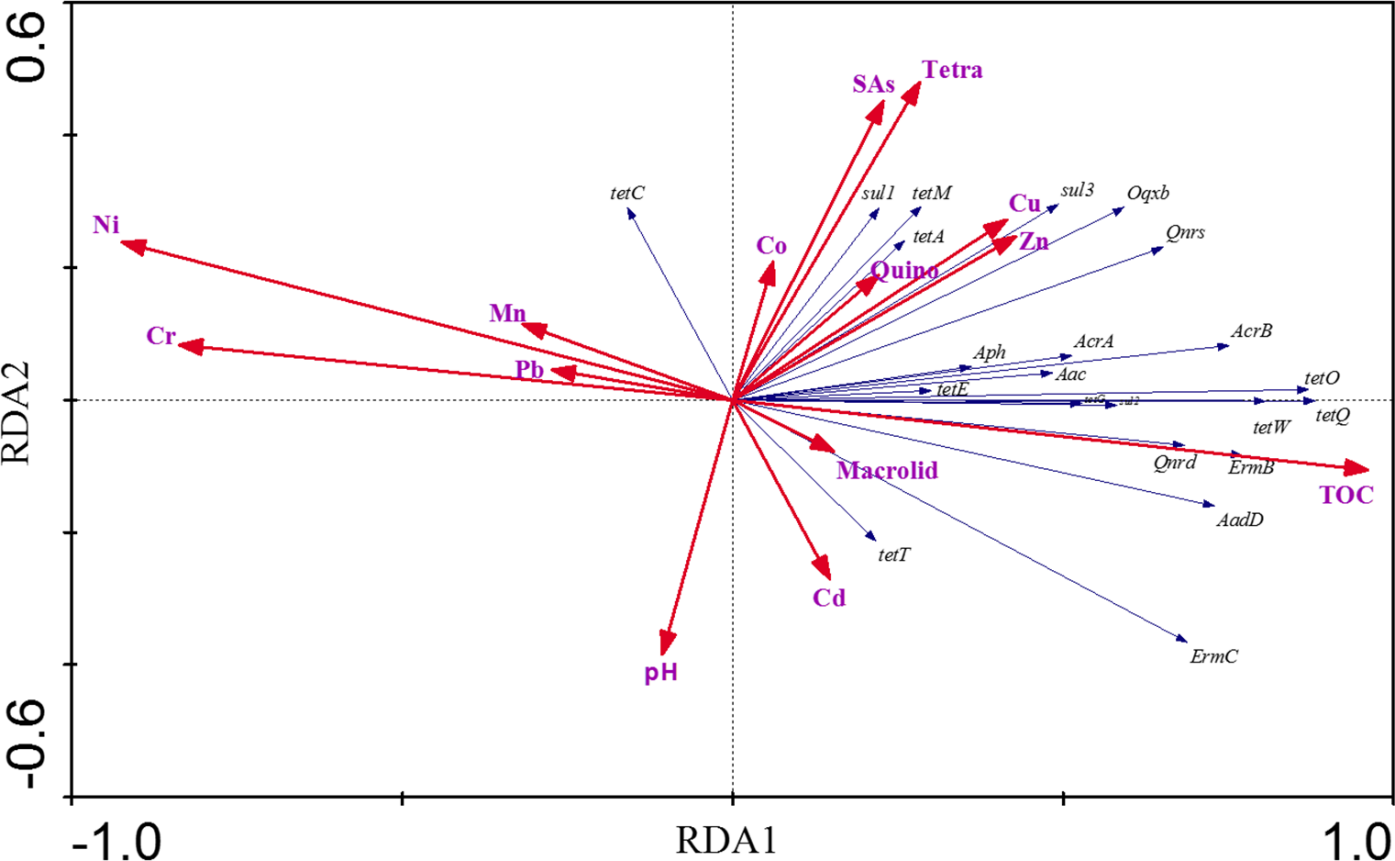
RDA results of absolute concentrations of 22 ARGs and environmental variables. (The angles between the arrows indicate the sign of the correlation between the ARGs and the associated environmental variables.)

From the published reference [25], it was interesting that the proliferation and spread effect of ARGs was more strongly driven by soil characteristics than by the source. Therefore, pearson correlation analysis was performed between every ARGs and environmental variables including antibiotics, heavy metals, and some physicochemical parameters (shown in Table 1). Furthermore, RDA analysis was conducted to identify the environmental factors that best influence the ARGs abundance (shown in Fig 6). The first two axes explained 61.9% of the cumulative percentage variance of the ARGs data with significance (p = 0.01). Based on both of the pearson correlation and RDA results, most ARGs showed significant positive relationships with environmental variables such as concentration of sulfonamides, tetracyclines, Cu, Zn and TOC.

**Table 1:** Correlations between the ARGs and environmental variables^#^.

	Quinolones	Sulfonamides	Tetracyclins	Macrolides	Cr	Mn	Co	Ni	Cu	Zn	Cd	Pb	TOC	pH
***tetA***		0.370**	0.328**		-0.343**			-0.314**	0.331**	0.484**		-0.368**	0.379**	
***tetC***		0.330**	0.298**						0.217*	0.303**				
***tetE***	0.243*		0.243*			-0.234*							0.293*	
***tetG***		0.251*	0.372**	0.319**	-0.483**		-0.234*	-0.507**	0.317**	0.553**		-0.371**	0.569**	
***tetM***		0.306**	0.405**		-0.440**			-0.443**		0.453**		-0.440**	0.373**	
***tetO***		0.429**	0.471**		-0.626**			-0.697**	0.531**	0.710**		-0.618**	0.721**	
***tetQ***		0.348**	0.404**		-0.665**	-0.226*		-0.726**	0.440**	0.694**		-0.678**	0.730**	
***tetT***				0.243*	-0.275**			-0.300**		0.240*		-0.235*	0.247*	
***tetW***		0.377**	0.415**		-0.594**		-0.209*	-0.678**	0.456**	0.612**		-0.617**	0.749**	
***sul1***		0.354**	0.507**		-0.395**	-0.211*		-0.462**		0.374**		-0.396**	0.445**	
***sul2***			0.381**	0.227*	-0.493**		-0.260*	-0.504**	0.208*	0.472**		-0.392**	0.553**	
***sul3***		0.335**	0.443**		-0.397**			-0.395**	0.396**	0.427**		-0.444**	0.469**	
***oqxb***		0.222*	0.548**		-0.391**			-0.406**	0.305**	0.335**		-0.497**	0.525**	
***qnrd***		0.342**			-0.529**		-0.259*	-0.561**	0.383**	0.573**		-0.467**	0.607**	
***qnrs***		0.322**	0.355**		-0.444**			-0.463**	0.334**	0.473**		-0.561**	0.557**	-0.278*
***ermB***		0.302**	0.391**	0.309**	-0.686**			-0.729**	0.377**	0.602**		-0.641**	0.693**	
***ermC***				0.209*	-0.520**	-0.251*	-0.227*	-0.611**		0.482**		-0.512**	0.571**	
***acrA***	-0.211*		0.331**		-0.641**	-0.228*		-0.615**		0.454**		-0.635**	0.601**	
***acrB***			0.348**		-0.592**	-0.275**		-0.617**	0.314**	0.540**		-0.633**	0.673**	
***aac***		0.289**	0.383**	0.209*	-0.452**		-0.275**	-0.503**	0.304**	0.473**		-0.473**	0.533**	
***aadD***		0.216*	0.312**	0.301**	-0.648**			-0.685**	0.338**	0.537**		-0.536**	0.610**	
***aph***		0.304**	0.347**		-0.408**			-0.488**	0.261*	0.551**		-0.436**	0.546**	

^#^ Values indicated the Pearson correlation coefficient (r), and all the detected data were obtained by three technical or biological replicates.

* Correlation is significant at the 0.05 level (2-tailed).

** Correlation is significant at the 0.01 level (2-tailed).

It was thought that antibiotics can provide selective pressure for the proliferation of resistant bacteria, through the horizontal transfer of ARGs or gene mutation [1] (Wu et al., 2010). However, there was no consistent research conclusion on the hypothesis that ARGs would be positively selected after exposure to antibiotics. Some researchers found strong correlation of ARGs and the corresponding antibiotics [26], but some studies reported no relationship of antibiotics and ARGs in environment [27] (Pei et al., 2006). Most studies found that this relashionship was not so strong, but statistical significant [1, 5], which can be explained that ARGs has different environmental fate and transport mechanisms [28]. The results in the present study showed that sulfonamides and tetracyclines may select for many ARBs, not only their corresponding ARGs, but macrolides may tend to select for its corresponding ARGs, *ermB* and *ermC*. Furthermore, the environmental degradation of antibiotics may also affect the correlation analysis results, that is, the antibiotics with long half-life, such as tetracyclines and sulfonamides, may often be found to correlate positively with the abundance of ARGs [29]. Meanwhile, there was nearly no correlation of ARGs and the antibiotics with low detected levels in the analysis, such as quinolones, possible attributed to the low usage of them.

Metals, such as copper (Cu), zinc (Zn), and arsenic (As), are commonly used in animal feeds as alternatives to antibiotics [25]. Most recent research announced that antibiotic resistance can be co-selected by metals [30–32], because both the antibiotics and heavy metal resistance genes are commonly found to be encoded on mobile genetic elements together, such as plasmids and transposable elements [33–36]. So the replacement of antibiotics with metals could actually make antibiotic resistance worse [25]. The results in the present study also reflected the characteristic that levels of Cu and Zn were positively correlated with ARGs significantly, because they were the frequent additives in the animal feeds. However, Cr, Mn, Pb and Ni showed negative relationships with ARGs, which may be attributed to the fact that the selection for ARGs under the exposure of heavy metals may occur more in gastrointestinal tracts of animals and in a manure waste holding system, so the relationship between feed additives (Cu and Zn) and ARGs was closer than that of heavy metals in environment (Cr, Mn, Pb and Ni).

For the other physicochemical parameters of soils, it was found that most ARGs was significantly correlated with total organic carbon (TOC), which was consistent with the conclusion of some researches by Wu et.al. and Su et.al. [1, 5]. However, there was no correlation of ARGs and soil pH, which was different from the reports that there was a negative relationship between *tet* genes and pH.

## Conclusion

Our study presentated the first-hand data on the pollution profile and spread characteristic of twenty-two ARGs conferring resistance to tetracyclines, sulfonamides, quinolones, aminoglycosides, and macrolides in all the relevant exposure matrices of sixteen animal farms in Southeastern China. Dominant ARGs in the environment were *sul*, *tet* and *erm* genes. The spread property of *sul* genes was most extensive and strong, followed by *tet* and *erm* genes. The significant difference was observed in the ARG levels of manure samples concerning different animal types, and the similar ARGs distribution rules were existing in poultry manure or porcine manure samples. The pollution of ARGs in the soils fertilized by poultry and cattle manures were more serious in northern Jiangsu, but no significant ARGs diversity was observed among porcine manured soils of five different regions. Meanwhile, ARGs distribution in soils also depended on the animal type of the livestock farms. Furthermore, most ARGs showed significant positive relationships with environmental variables such as concentration of sulfonamides, tetracyclines, Cu, Zn and TOC.

## Supporting Information

S1 Fig Mean concentration and detection frequency of each ARGs in manure samples. (TIF)

S2 Fig Mean concentration of 6 classes of ARGs in soil samples (*tet*: tetracyclin resistance genes; *sul*: sulfonamide resistance genes; *qnr*: quinolone resistance genes; *erm*: macrolide resistance genes; *acr*: multidrug resistance genes; *ami*: aminoglycoside resistance genes). (TIF)

S1 Table Basic information of the sampling livestock farms. (PDF)

S2 Table Information of primers for qPCR. (PDF)

S3 Table Equations of standard curves. (PDF)
