# Supplementary figures and images for: A Comprehensive Analysis on Spread and Distribution Characteristic of Antibiotic Resistance Genes in Livestock Farms of Southeastern China
Source: PLoS One. 2016 Jul 7;11(7):e0156889. doi: 10.1371/journal.pone.0156889 (PMC4936668; doi:10.1371/journal.pone.0156889)

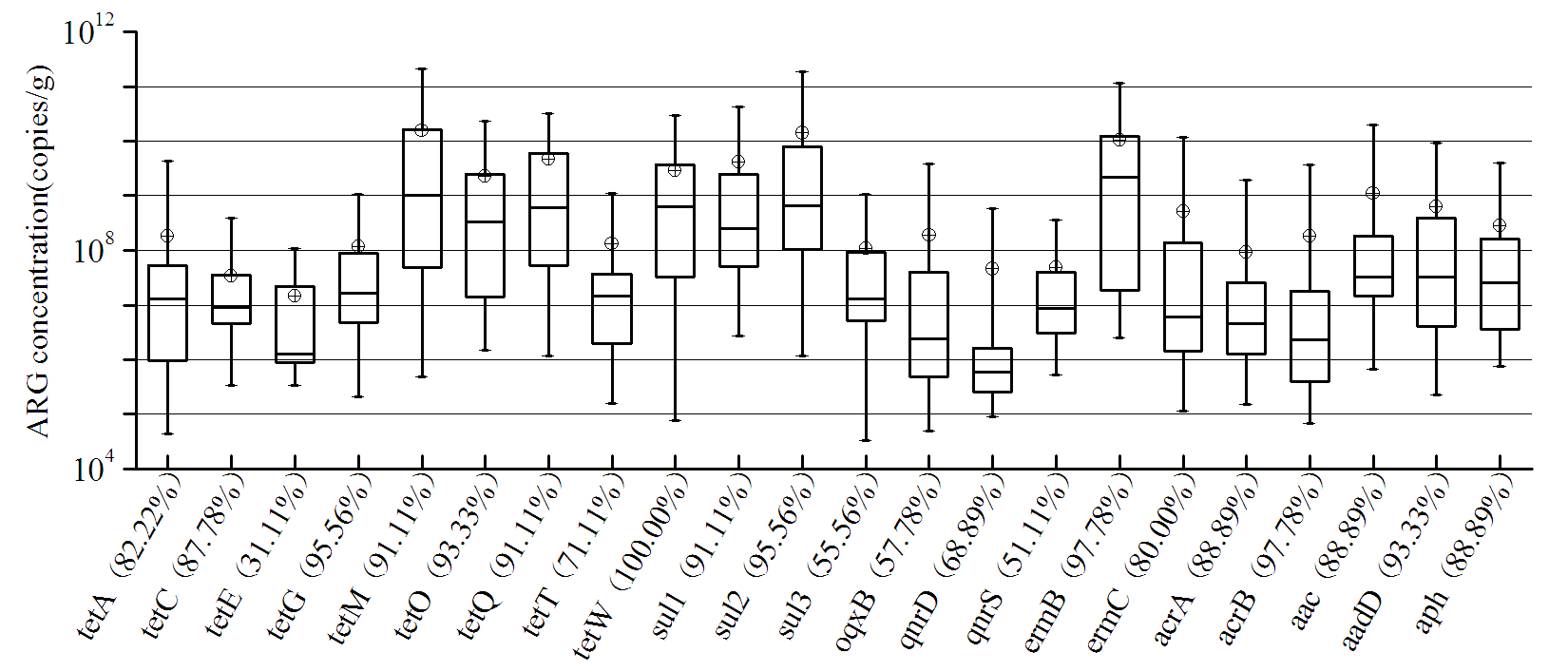

Supplement: S1 Fig — (TIF) [file pone.0156889.s001.TIF]

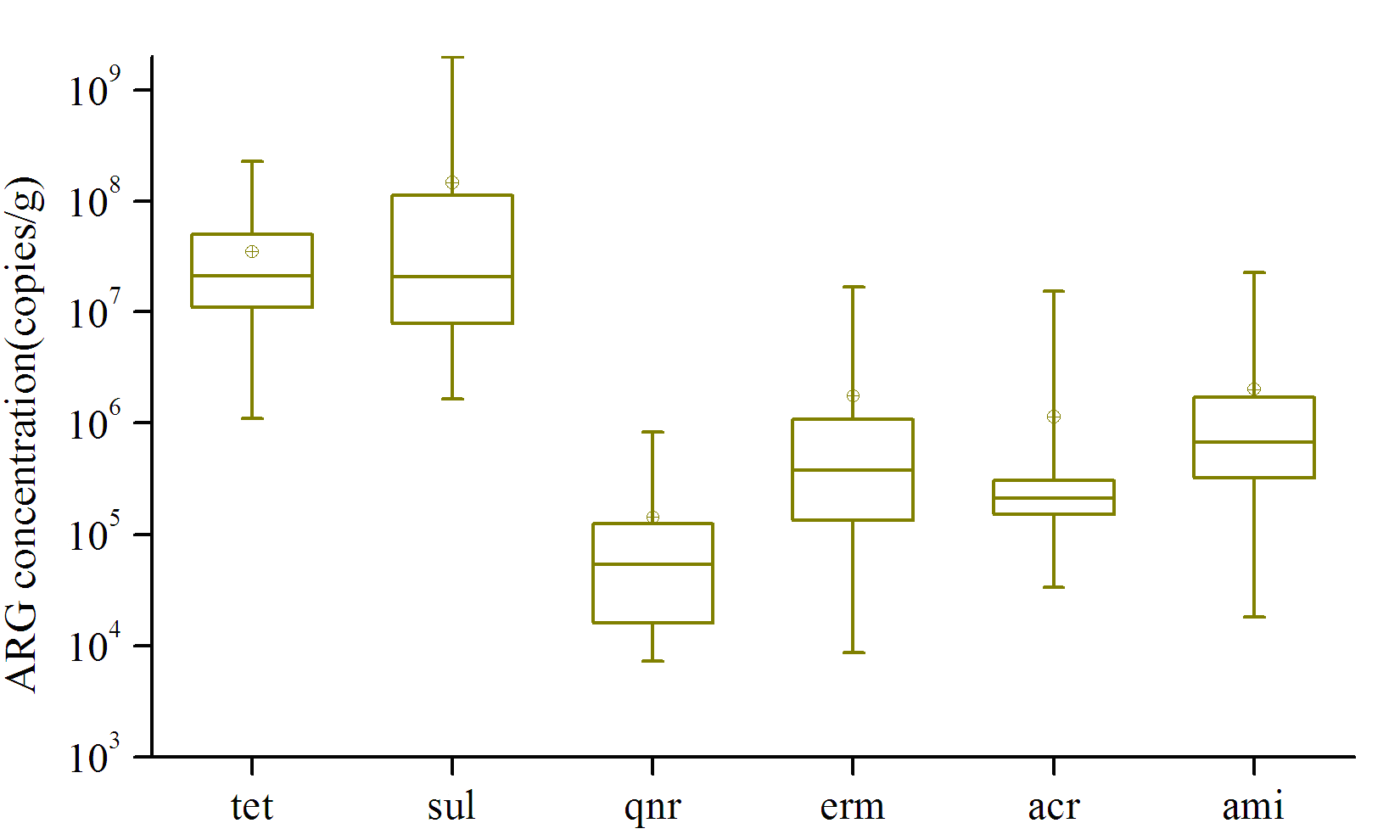

Supplement: S2 Fig — (TIF) [file pone.0156889.s002.TIF]
