# Supplementary material for: A Comprehensive Analysis on Spread and Distribution Characteristic of Antibiotic Resistance Genes in Livestock Farms of Southeastern China
Source: PLoS One. 2016 Jul 7;11(7):e0156889. doi: 10.1371/journal.pone.0156889 (PMC4936668; doi:10.1371/journal.pone.0156889)
Supplement: S1 Table — (PDF) [file pone.0156889.s003.pdf]

S1 Table Basic information of the sampling livestock farms

NO	livstock type	locati on	sampling time	Latitude and longitude	Scale	Numbers of manure samples collected	Numbers of soil samples collected	the last fertilization time	the way the manure treated
1	chicken	CZ	2014.4	N31°40'33 "，E119°38'36"	50000	3	2	2014,2	deliver to the organic fertilizer plant
2	cattle	CZ	2014.4	N31°41'55"，E119°49'4"	1000	3	3	2014,1	biogas fermentation
3	pig	CZ	2014.4	N31°41'55"，E119'49'4"	200	3	3	2013,11	biogas fermentation
4	chicken	NJ	2014.4	N32°29'44"，E118°40'05"	20000	2	4	2013,10	fish feed， deliver to the organic fertilizer plant
5	cattle	NJ	2014.3	N32°30'1"，E118°40' 54"	3000	7	3	2013,10	separation into wet and dry,dry-compost-fertilization, liquid- lagoon
6	pig	NJ	2014.3	N32°30'32"，E118°40' 55"	4000	8	3	2013,10	compost， deliver to the organic fertilizer plant
7	chicken	HA	2014.4	N32°33'57.2"，E120°35'33.7"	270000	2	-	-	deliver to the organic fertilizer plant
8	cattle	HA	2014.4	N32°32'29.6"，E120°36'46.4"	200	3	3	-	separation into wet and dryliquid-fertilization
9	pig	HA	2014.4	N32°37'54.3"，E120°25'54.1"	20000	5	3	-	biogas fermentation

10	chicken	XZ	2014.4	N34°23'29.5"，E118°25'13.5"	400000	2	3		deliver to the organic fertilizer plant
11	cattle	XZ	2014.4	N32°30'1"，E118°40' 60"	800	3	3	-	deliver to the organic fertilizer plant
12	pig	XZ	2014.4	N34°22'11.7"，E118°32'21.8"	8000	4	3	-	biogas fermentation
13	chicken	SQ	2014.4	N34°22'38.7"E 118°12'21"	60000	2	3	-	compost
14	meat chicken	SQ	2014.4	N33°42'39.2"E 118°12'47.2"	3000	1	6	-	compost
15	cattle	SQ	2014.4	N33°42'23.1"E 118°12'42.3"	500	2	3	-	deliver to the organic fertilizer plant
16	pig	SQ	2014.4	N33°41'14.4"E 118°11'29.6"	50	3	5	-	compost
