# Supplementary material for: A Comprehensive Analysis on Spread and Distribution Characteristic of Antibiotic Resistance Genes in Livestock Farms of Southeastern China
Source: PLoS One. 2016 Jul 7;11(7):e0156889. doi: 10.1371/journal.pone.0156889 (PMC4936668; doi:10.1371/journal.pone.0156889)
Supplement: S2 Table — (PDF) [file pone.0156889.s004.pdf]

S2 Table Information of primers for qPCR

Gene	sequence (5’>3’)	annealing temperature (℃)	product size (bp)
*tet*A	F:GCTACATCCTGCTTGCCTTC<br>R:CATAGATCGCCGTGAAGAGG	60	210
*tet*C	F:TGCAACTCGTAGGACAGGTG<br>R:ACCAGTGACGAAGGCTTGAG	60	139
*tet*E	F:ACTGTGATGATGGCACTGGT<br>R:CTGCAACATTAGCCTTTCCA	57	112
*tet*G	F: CTCGGTGGTATCTCTGCTCA<br>R: CAGAACGAATGGTTTGATGC	58	147
*tet*M	F:ACAGAAAGCTTATTATATAAC<br>R:GGCGTGTCTATGATGTTCAC	51	171
*tet*O	F:ACGGARAGTTTATTGTATACC<br>R:TGGCGTATCTATAATGTTGAC	52	171
*tet*Q	F:AGAATCTGCTGTTTGCCAGTG<br>R:CGGAGTGTCAATGATATTGCA	60	167
*tet*T	F :AGAGTCGACAATGCTACAACGA<br>R :TGAAACTGTAGACGCCCGTA	56.1	84
*tet*W	F:GAGAGCCTGCTATATGCCAGC<br>R:GGGCGTATCCACAATGTTAAC	60	168
*sul*1	F:TGTCGAACCTTCAAAAGCTG<br>R:TGGACCCAGATCCTTTACAG	60	113
*sul*2	F:ATCTGCCAAACTCGTCGTTA<br>R:CAATGTGATCCATGATGTCG	60	89
*sul*3	F: AGGCTTGGCAAAGTCAGATT<br>R: CACCAGCCTCAACTAAAGCA	57	152
*qnr*D	F:ACGACAGGAATAGCTTGGAAGG<br>R :TCAGCCAAAGACCAATCAAACG	57	373
*qnr*S	F: TAAATCACACGCACGGAACT<br>R: AACAGGGTGATATCGAAGGC	55.3	134
*oqx*B	F:TCCTGATCTCCATTAACGCCCA<br>R:ACCGGAACCCATCTCGATGC	60	131
*erm*B	F: AGGGTTGCTCTTGCACACTC<br>R: CTGTGGTATGGCGGGTAAGT	58	119
*erm*C	F:GAAATCGGCTCAGGAAAAGG<br>R:TAGCAAACCCGTATTCCACG	56	292
*acr*A	F:CTCTCAGGCAGCTTAGCCCTAA<br>R:TGCAGAGGTTCAGTTTTGACTGTT	60	107
*acr*B	F:GGTCGATTCCGTTCTCCGTTA<br>R:CTACCTGGAAGTAAACGTCATTGGT	60	105
*aad*D	F:ATGGGGATGATGTTAAGGCT<br>R:TCACTTCCACCTTCCACTCA	55	153
aph	F:ATTCAACGGGAAACGTCTTG<br>R:ACGCTACCTTTGCCATGTTT	56	173
aac	F:GGAAGAAGAAACCGATCCAG<br>R:CAACTCAACCAGAGCTCGAA	55	109
16S rDNA	F:CAATGGACGAAAGTCTGACG<br>R:ACGTAGTTAGCCGTGGCTTT	60	146
