# Supplementary material for: A Comprehensive Analysis on Spread and Distribution Characteristic of Antibiotic Resistance Genes in Livestock Farms of Southeastern China
Source: PLoS One. 2016 Jul 7;11(7):e0156889. doi: 10.1371/journal.pone.0156889 (PMC4936668; doi:10.1371/journal.pone.0156889)
Supplement: S3 Table — (PDF) [file pone.0156889.s005.pdf]

S3 Table Equations of standard curves

Gene	equations	R^2
*su1*1	Y=-3.3959X+41.620	0.9965
*sul*2	Y=-3.2854X+41.418	0.9955
*sul*3	Y=-3.4217X+38.945	0.9965
*tet*A	Y=-3.2066X+36.096	0.9909
*tet*C	Y=-3.4240X+38.739	0.9940
*tet*E	Y=-3.4168X+39.296	0.9989
*tet*G	Y=-3.4469X+37.547	0.9996
*tet*T	Y=-3.4388X+39.398	0.9998
*tet*W	Y=-3.3614X+37.913	0.9995
*tet*O	Y=-3.3739X+37.593	0.9944
*tet*Q	Y=-3.3424X+36.347	0.9929
*tet*M	Y=-3.3078X+40.105	0.9939
*acr*A	Y=-3.2525X+37.686	0.9987
*acr*B	Y=-3.3674X+37.552	0.9965
*qnr*S	Y=-3.4308X+38.742	0.9995
*qnr*D	Y=-3.4280X+37.994	0.9978
*oqx*B	Y=-3.4174X+37.796	0.9997
*erm*B	Y=-3.4059X+39.630	0.9995
*erm*C	Y=-3.4181X+37.882	0.9999
*aad*D	Y=-3.3724X+37.750	0.9984
aph	Y=-3.2845X+37.029	0.9959
aac	Y=-3.4068X+38.206	0.9953
16S rDNA	Y=-3.3456X+38.357	0.9993
